# Gardening in the desert: a spatial optimization approach to locating gardens in rapidly expanding urban environments

**DOI:** 10.1186/s12942-017-0110-z

**Published:** 2017-10-16

**Authors:** Elizabeth A. Mack, Daoqin Tong, Kevin Credit

**Affiliations:** 1 Department of Geography, Environment and Spatial Sciences, Michigan State University, Geography Building, 673 Auditorium Rd, Room 202, East Lansing, MI 48824 USA; 2 School of Geographical Sciences and Urban Planning, Arizona State University, Tempe, AZ 85281 USA

**Keywords:** Community gardens, Vacant land, Spatial optimization, Food access, Food deserts, Urbanization, Urban agriculture

## Abstract

**Background:**

Food access is a global issue, and for this reason, a wealth of studies are dedicated to understanding the location of food deserts and the benefits of urban gardens. However, few studies have linked these two strands of research together to analyze whether urban gardening activity may be a step forward in addressing issues of access for food desert residents.

**Methods:**

The Phoenix, Arizona metropolitan area is used as a case to demonstrate the utility of spatial optimization models for siting urban gardens near food deserts and on vacant land. The locations of urban gardens are derived from a list obtained from the Maricopa County Cooperative Extension office at the University of Arizona which were geo located and aggregated to Census tracts. Census tracts were then assigned to one of three categories: tracts that contain a garden, tracts that are immediately adjacent to a tract with a garden, and all other non-garden/non-adjacent census tracts. Analysis of variance is first used to ascertain whether there are statistical differences in the demographic, socio-economic, and land use profiles of these three categories of tracts. A maximal covering spatial optimization model is then used to identify potential locations for future gardening activities. A constraint of these models is that gardens be located on vacant land, which is a growing problem in rapidly urbanizing environments worldwide.

**Results:**

The spatial analysis of garden locations reveals that they are centrally located in tracts with good food access. Thus, the current distribution of gardens does not provide an alternative food source to occupants of food deserts. The maximal covering spatial optimization model reveals that gardens could be sited in alternative locations to better serve food desert residents. In fact, 53 gardens may be located to cover 96.4% of all food deserts. This is an improvement over the current distribution of gardens where 68 active garden sites provide coverage to a scant 8.4% of food desert residents.

**Conclusion:**

People in rapidly urbanizing environments around the globe suffer from poor food access. Rapid rates of urbanization also present an unused vacant land problem in cities around the globe. This paper highlights how spatial optimization models can be used to improve healthy food access for food desert residents, which is a critical first step in ameliorating the health problems associated with lack of healthy food access including heart disease and obesity.

**Electronic supplementary material:**

The online version of this article (doi:10.1186/s12942-017-0110-z) contains supplementary material, which is available to authorized users.

## Introduction

The World Bank notes that developing countries have large amounts of unused land, which run the risk of marginalizing a growing number of urban poor [1]. Cities in countries around the globe including Afghanistan [2], India [3] and Brazil [4] are urbanizing rapidly and experiencing symptoms of rapid growth including lack of food access and unused vacant land. Urban agriculture initiatives are a promising solution to the vacant land and food security problem in global cities, and urban residents around the world are pursuing urban gardening initiatives [5]. These gardening initiatives are not only important for establishing communities that are more connected and have better access to food systems, they also represent an important piece of the puzzle in solving the growing global health issue of obesity given the link between lack of access to quality food and health [6–9].

The United Nations estimates that in 2014, 54% of the world’s population lived in urban areas, and this number is projected to increase to 66% by 2050 [10]. Rising rates of urbanization mean diminished connections to food sources as agricultural land disappears [3] and local food sources disappear in favor of superstores that meet consumer demand for standardized, unblemished food products [11–13]. The shift in size, scale, and location of food outlets over the past 60 years—from small, urban neighborhood stores to large suburban superstores—is a global phenomenon that is increasingly prevalent in the food economics of the developed world [12]. Locales where residents do not have access to and/or cannot afford healthy food are commonly referred to as “food deserts”.

While there has been a wealth of research dedicated to understanding the location of food deserts [14, 15] and the benefits of urban gardens [16–18] few studies have linked these two strands of research together to analyze whether urban gardening activity may be a step towards addressing issues of food access for residents of food deserts. To better understand the neighborhood context of urban gardening activity and its spatial linkages with food deserts, this study analyzes the locations of food deserts and urban gardening activity. The key contribution of the study is the use of a garden siting technique, the maximal covering location model, to propose alternative urban garden sites and improve food access for area residents. The potential utility of this type of analytical approach is demonstrated for Phoenix, Arizona, which is rapidly urbanizing and has a vacant land problem. This technique can be applied however to any urban environment where the necessary data are available. In this respect, siting gardens on vacant land is a particularly promising tool for improving food access and urban food security in cities around the globe.

## Background: food access and food deserts

Food access is a precursor to healthy food consumption and healthy food consumption is associated with better health [19–22]. While the food environment is not the sole driver of food consumption practices, studies do find linkages between healthy food access and the quality of human health [7, 8, 23, 24]. Given the health implications associated with food access, several studies have endeavored to identify neighborhoods, especially low-income neighborhoods, with inadequate access to healthy food [25]. These studies find that changes in food retailing practices, with small independent retailers slowly replaced by large superstores, have changed the landscape of food access [26, 27], leaving urban residents with fewer food choices. This retailing change makes suburban locations more attractive because of the land area required for larger stores and the reduced expense of land in suburban areas [26]. It is important to note that this consolidation of food outlets also impacts rural residents when local neighborhood stores close due to competition from larger retailers [15]. While a majority of the literature on food deserts emphasizes this issue in an urban context [28–30], more recent work has uncovered that the hinterlands of metropolitan areas have residents that suffer from lack of access to healthy food [25], as well as residents in suburban [31] and rural areas [15, 26, 32]). Sharkey et al. [33] note that food access in rural locations is particularly important to analyze given the compounding challenges of distance and transportation access in rural environments. Work also highlights the importance of considering temporal aspect of food access related to changes in public transportation schedules and the operating hours of food stores [34]. Farber, Morang, and Widener [35], note that the operating hours of public transportation can impact travel times, which then impacts peoples’ ability to patronize food outlets.

Despite the amount of attention dedicated to food access, there is a lack of consensus on the definition of food deserts [26, 36, 37]. Table 1 provides several examples of food desert definitions, and highlights the sources of variation in how these are defined. Some definitions define a particular distance that constitutes good food access [14, 38, 39]. Some definitions explicitly refer to low-income neighborhoods or groups [15, 30, 40] while others do not [14, 38]. Other sources of variation in food desert definitions include the explicit mention of transit times [41] and/or specific mention of a particular type of food outlet used to determine food access.

**Table 1:** Definitions of food deserts used in previous studies

Definition	Geography	Study
Term *first used* in UK to describe “rapidly decreasing number of grocers in urban, low income neighborhoods after World War II”	Urban areas	[40, p. 3]
Spatial disparity in access to retail food stores	Urban areas	[82]
Areas “where cheap and varied food is only accessible to those who have private transport or are able to pay the costs of public transport”	Urban areas	[83, p. 65]
Areas with barriers to food access based on “ability” (physical barriers), “assets” (financial barriers), or “attitudes” (state of mind)	Urban areas	[84, p. 241]
“Economic and physical access constraints perceived and experienced by disadvantaged consumers in an area of compound social exclusion and poor food retail access”	Urban areas	[85, p. 2084]
Empirical definition—minority neighborhoods with lower access to healthy food destinations within 5-min travel times	Urban areas	[41]
“Places where the transportation constraints of carless residents combine with a dearth of supermarkets to force residents to pay inflated prices for inferior and unhealthy foods at small markets and convenience stores”	Urban areas	[44, p. 352]
“Socially-distressed neighbourhoods with relatively low average household incomes and poor access to healthy food”	Urban areas	[30, p. 1]
“Urban areas with 10 or fewer stores and no stores with more than 20 employees”	Urban areas	[29, p. 372]
“Poor urban areas, where residents cannot buy affordable, healthy food”	Urban areas	[76, p. 436]
Locales situated more than 10 miles (16 km) from a supermarket	Rural	[14, 38]
“Socio-economically disadvantaged areas with relatively low household incomes and poor geographical access to nutritious, affordable food sources”	Not specified	[15, p. 2]
“Areas of relative exclusion where people experience physical and economic barriers to accessing health food”	Not specified	[27, p. 138]
A low-income tract where at least 33% of the population is greater than 1 mile (1.61 km) (in an urban area) or greater than 10 miles (16 km) (in a rural area) from the nearest supermarket, supercenter, or large grocery store	Urban and rural areas	[39]
“Low-income, urban neighborhoods, often centrally located, with inadequate physical or economic access to healthy food”	Urban areas	[25, p. 204]

In addition to variations in food desert locations and counts stemming from basic definitional issues, Bao and Tong [42] point out inconsistencies in the findings of food desert studies that are related to differences in the spatial scale and level of data aggregation. Studies have also found that the choice of study area matters, and that not all locations have a food desert problem. For example, Apparicio et al. [43] found no evidence of a food desert problem in Montréal, which suggested the need for other mechanisms beyond improved healthy food access to resolve diet-related health problems for Montréal residents.

## Background: urban gardens

Several studies of alternative means of food access have analyzed small food stores as a means of solving the food desert problem [6, 41, 44]. Mobile vans have also been suggested as a means of providing food insecure neighborhoods with fresh fruits and vegetables [45]. Other studies have suggested that building a strong local food economy through farmer’s markets and direct sales from farms could be an important strategy in the fight against obesity [46]. This approach includes the use of community gardens as a mechanism for providing access to nutritious foods [47]. Locally grown food has a long history as an alternative means of food access in urban environments, and studies have noted that in the United Kingdom and the United States, gardens are a notable feature of the urban landscape, although the intensity of gardening activities varies over time [48].

Throughout the history of the United Kingdom, allotment gardens served as an important source of employment and food [48]. In the United States, urban gardens were part of the social reform movements in the 1890s, and were also an important source of food during the Great Depression [49, 50]. In both World Wars, urban gardens served as an alternative food source. During World War II in particular, “victory gardens” were an important source of fresh food for U.S. residents so food stuffs could be sent to troops abroad [49]. Post-WWII, urban gardening efforts experienced a comparative lull until the 1970s, when gardens become a component of urban revitalization efforts [49]. Starting in the 1970s, federal programs such as the United States Department of Agriculture’s (USDA) Urban Garden Program continued to support gardening activities in urban environments [48]. Today, in cities around the globe—from Puerto Maldonado, Peru to Canberrra, Australia to Mumbai, India—organizations and urban residents are now growing food in urban environments [5].

Because of rising rates of urbanization and growing interest in urban food production globally, studies have begun to incorporate farmer’s markets and community gardens into analyses of food deserts. It has been noted that studies that do not consider these sources of fresh foods will overestimate inequities in food access [51]. Studies have also found that community gardens are a viable source of food for low-income people and can provide additional benefits to neighborhoods by improving the attitudes and outlooks of residents [16, 17]. As regards access and consumption of healthy food, Litt et al. [17] found that community gardeners were more likely to consume fruits and vegetables than were home gardeners and non-gardeners.

Given the importance of local, healthy food sources, researchers have also begun to examine the potential for cultivating food within urban environments [52, 53]. These studies use a wide range of tools including geographic information systems (GIS), remote sensing, and site suitability techniques. For example, Kremer and DeLiberty [52] combined GIS and remote sensing techniques to examine the availability of urban land in Philadelphia, Pennsylvania for garden activity. Site suitability analysis was used to propose locations for urban gardens in cities ranging from Hanoi, Vietnam [54] and Chittendon County, Vermont [55]. In Portland, Oregon and Vancouver, Canada, Mendes et al. [56] conducted a visual assessment of parcels, including tree canopy and built environment characteristics, to identify the most suitable government-owned land on which to pursue urban agriculture projects. Finally, participatory mapping has been used to visualize relationships in local food systems [57] and locate healthy food retail outlets [58]. This approach draws upon the knowledge of experts to understand and restructure aspects of local food systems.

While these techniques represent important advancements to understanding and improving urban food systems, they are not without drawbacks. Studies have found that remote sensing techniques do not accurately identify garden locations because of their small size and heterogeneous layouts, which produce non-uniform visual patterns [53]. Site suitability techniques are an improvement over remote sensing techniques because they are capable of incorporating multiple variables above and beyond land use, but are perhaps more accurately viewed as an initial screening process that helps to find suitable areas for gardens. From this perspective, spatial optimization models represent a potential improvement over site suitability analyses. This brand of optimization model can be viewed as a type of site selection analysis with additional considerations, that include: (1) the number of gardens to site due to budget constraints, (2) a more accurate way to account for multiple factors, and (3) the spatial relationship among gardens (and between neighborhoods and gardens). Thus, spatial optimization models not only have the site-identifying capacity of site suitability analyses, but they also have the added capability of providing information about the spatial configuration of sites, in conjunction with a sense of tradeoffs about the number of gardens to be cited and the population of interest serviced by these gardens. Because of the enhanced analytical capabilities of these models, they can be used to analyze how urban gardens may be distributed better to resolve issues of access for food desert residents.

## Methods

### Study area

**Fig. 1:**
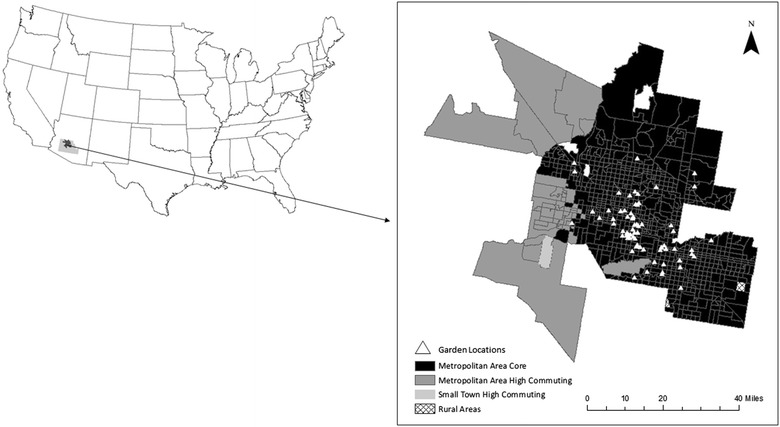
Urban–rural classification of Phoenix, Arizona census tracts

Maricopa County, which contains the majority of the Phoenix metropolitan area, is the study area for this analysis. Figure 1 depicts the distribution of urban, suburban, and rural areas across the metropolitan area. Dark colors represent the most tracts while lighter colors represent comparatively rural tracts. These 2010 Rural–Urban Commuting Area (RUCA) categories of the urban–rural continuum were obtained from the United States Department of Agriculture (USDA) and contain 10 categories of tracts ranging from the most urbanized (code 1) to the most rural tracts (code 10). Based on this classification scheme, the majority of tracts (95%) across Phoenix are classified as part of the metropolitan area core. Only two tracts are classified as rural. Interestingly, tracts that are classified as having high levels of commuting to the urban core are located mostly in the West Valley of Phoenix in communities such as Glendale, El Mirage, and Surprise.

These high commuting tracts highlight the sprawling nature of the metropolitan area [59, 60], which means that residents are more likely to drive to everyday activities than residents in older, more walkable metropolitan areas. In this context, several locations across Maricopa County represent less centrally located communities, where issues of adequate access to healthy foods are perhaps exacerbated [25]. This issue of sprawl is not unique to Phoenix but is characteristic of cities across the globe. Another feature of the metropolitan area that is characteristic of rapidly urbanizing cities is a vacant land problem [61–63] with over 10,000 acres of unused land [63]. While a lot of this land is on the urban fringe, satellite imagery also highlights many examples of vacant lots in built-up portions of the study area. Recently, City of Phoenix officials have attempted to find temporary uses for vacant land and community gardens represent one of these proposed land uses [61]. For example, as part of the Phoenix Renews project, a 15-acre vacant lot at the intersection of Central Avenue and Indian School Road was proposed as the location of an urban community farm. Unfortunately, the owner of the lot defaulted on payments and had to return the land to the U.S. Department of the Interior [64]. This closure means that local gardeners who started growing crops will lose their plots, and must find a location elsewhere. Given the potential for gardens to alleviate poor access to healthy foods, finding suitable locations for community gardens is no easy task, but perhaps a necessary step to move towards a more comprehensive resolution to the vacant land problem in Phoenix, and to simultaneously improve food access for residents.

### Data

Given the complex swathe of factors to consider in siting gardens, this study will analyze current sites of urban gardening activities with an emphasis on their neighborhood context. It will then propose new locations for urban gardening activity to improve access for food desert residents. To provide a more comprehensive perspective on urban garden locations, several variables are used to characterize the neighborhood environment. To do this, a variety of data including housing, land use, zoning, demographic, and socio-economic characteristics were collected at the census tract level based on the precedent of prior studies [25, 30, 43]. From this perspective, special attention was devoted to collecting information about economic disadvantage given the link between socio-economic status (SES) and access to healthy food [25, 28, 39]. Table 2 contains summary information about these data.

**Table 2:** Description of data and data sources

Variable	Description	Data source
Land use	Parcel data about land use data for 2014 in 16 categories: i.e., industrial, single family residential, commercial, office	Maricopa Association of Governments (MAG)
Food desert	Tract level data about food access reported in 2013	United States Department of Agriculture (USDA)
Median home value	Median value of owner-occupied housing units (current dollars)	2014 American Community Survey: 5-year estimates (2010–2014)
Percent owner occupied	Percent of housing units that are owner occupied	2014 American Community Survey: 5-year estimates (2010–2014)
Percent vacant housing units	Percent of housing units that are vacant	2014 American Community Survey: 5-year estimates (2010–2014)
Median contract rent	Renter-occupied housing units paying cash rent (current dollars)	2014 American Community Survey: 5-year estimates (2010–2014)
Percent Black	Percent of the population that is Black	2014 American Community Survey: 5-year estimates (2010–2014)
Percent Hispanic	Percent of the population that is Hispanic	2014 American Community Survey: 5-year estimates (2010–2014)
Percent bachelor’s	Population aged 25 and older with a bachelor’s degree or higher	2014 American Community Survey: 5-year estimates (2010–2014)
Percent food stamps	Percent of households receiving Food Stamps/SNAP in the past 12 months	2014 American Community Survey: 5-year estimates (2010–2014)
Percent no healthcare	Percent of the population with no healthcare	2014 American Community Survey: 5-year estimates (2010–2014)
Percent under 18 with no healthcare	Percent of the population under 18 with no healthcare	2014 American Community Survey: 5-year estimates (2010–2014)
Percent unemployment	Percent of the population 16 years and older that is unemployed	2014 American Community Survey: 5-year estimates (2010–2014)
Food outlets	2010 point level food outlet data aggregated to census-tracts	ESRI Reference USA
Percent of workers who drove alone to work	Percentage of workers 16 and over who drove alone to work in tract	2014 American Community Survey: 5-year estimates (2010–2014)
Percent of workers commuting using non-auto modes	Percentage of workers 16 and over who commuted to work using public transit, bicycle, or walking in tract	2014 American Community Survey: 5-year estimates (2010–2014)
Less than 15 min travel time to work	Percentage of commuters with a commute time of less than 15 min in tract	2014 American Community Survey: 5-year estimates (2010–2014)
30 min or more travel time to work	Percentage of commuters with a commute time of greater than 30 min in tract	2014 American Community Survey: 5-year estimates (2010–2014)

### Garden data

Urban garden locations are derived from a list obtained from the Maricopa County Cooperative Extension (MCCE) office at the University of Arizona which provided the name and address for gardens across the county. Information from this database was verified from aerial imagery on Google Maps, which provided historical images of garden locations in some cases. When necessary, contacts with garden managers were also used to verify the start and end date of the gardens to ascertain whether they were active or inactive. The address of active gardens was also verified because some gardens had moved since their initial start date. When garden managers could not be contacted, in-person visits were made to the address for the garden listed in the database to verify the status of the garden. Above and beyond information in this database, efforts were made to triangulate and supplement data from the MCCE list with information from the American Community Garden Association (ACGA) website and city government websites. Out of the 99 garden locations identified, 77 gardens locations were verified within the boundaries of the Phoenix metropolitan area. Of these 77 gardens, 68 were active at the time the data were collected. However, both active and inactive gardens will be used in the analysis that follows to understand both past and current trends in garden locations given the transient nature of urban gardening activity [49].

Once the addresses of garden locations were verified, they were geocoded and matched to their relevant census tract in order to integrate garden data with data collected from other sources. Census tracts were then assigned to one of three categories: tracts that contain a garden, tracts that are immediately adjacent to a tract with a garden, and all other non-garden/non-adjacent census tracts. The adjacency category was used to identify tracts that are proximal to a tract with a garden, as opposed to a binary breakdown of tracts into those with and without a garden. This category is important to consider because these tract residents are still nearby a source of fresh fruits and vegetables. In the analysis that follows, 75 of the 77 garden sites were located in Census tracts that fell within the boundaries of Phoenix area neighborhoods. Thus, these 75 gardens will serve as the basis for the ANOVA comparison of garden-oriented neighborhoods and non-garden oriented neighborhoods. For the spatial optimization analysis, all 77 gardens will be used because the analysis assigns gardens to tracts based on a threshold distance of 1 mile (1.61 km).

### Food outlet and food desert information

In addition to information about garden locations, healthy food outlet information from the ESRI Reference USA dataset was compiled using the definition of food outlets from Raja et al. [41]. Based on this study, point-level information about outlets selling healthy food was compiled and aggregated to census tracts. These data include the following types of food outlets: supermarkets, natural food stores, meat and fish stores, specialty food stores, and fruit and vegetable stores.^1^ Bakeries and dairy stores were excluded from the analysis because their food offerings could not be classified as healthy: most of the dairy stores in this database were verified as selling frozen yogurt.

Census tract information about food deserts was obtained from the United States Department of Agriculture (USDA). Since the USDA provides several definitions of food deserts, the definition used in this study defines food deserts as Census tracts with low access to supermarkets or larger grocery stores where low access means residents are more than 1 mile (1.61 km) from food outlets in urban areas and more than 10 miles (16.09 km) from food outlets in rural areas [65].

### Demographic and socio-economic data

Contextual information about the demographic and socio-economic profile of Phoenix area residents was compiled from the National Historic Geographic Information System (NHGIS) Database, which contains American Community Survey (ACS) estimates for census tracts between 2010 and 2014. Demographic information collected from this database includes information about race/ethnicity, educational attainment, as well as the poverty status and income level of households.

### Housing, land use and zoning information

Housing and land use information were also collected to provide a sense of the types of housing and land uses in and around tracts with gardens. Information about home value and occupancy status were obtained from the NHGIS archive of ACS data 2010–2014 5-year estimates. Parcel level information about land use across the metropolitan area was obtained from the Maricopa Association of Governments (MAG) database as of 2014. A critical aspect of this database is the information about vacant developable land, which is important to identify given the vacant land problem discussed above, and because these vacant land parcels represent potential urban garden locations. Parcel data were aggregated to the census tract level to get a sense of the amount of a particular land use (in square miles) within each census tract. To incorporate information about travel time for residents, tract-level data from the ACS 2010–2014 5-year estimates on commuting mode and travel time to work were also gathered.

### Analytical approach

Analysis of variance (ANOVA) is used to determine whether there are statistical differences between the three categories of tracts described above (contain a garden, adjacent to a garden, not adjacent/does not contain a garden) based on the contextual data summarized in Table 2. This portion of the analysis is needed to test the following three hypotheses:

#### **H1**

Households in tracts with a garden, or nearby a garden, will have higher socioeconomic status than households in tracts without gardens.

#### **H2**

Tracts with gardens, or nearby a garden, will have different land uses than tracts without a garden.

#### **H3**

Tracts without gardens will have poor access to other types of food sources than tracts with gardens, or nearby a garden.

These hypotheses are important to test, because they can help characterize important economic, land use and food access differences between the three types of tracts. If for example, there are no differences in food access between the three categories of tracts, a reconfiguration of current garden locations is not necessary to improve access for residents.

After analyzing the neighborhood context of urban gardens, location models are used to identify potential sites for future garden activity. Here, it is important to remember that this analytical approach is different from prior remote sensing and site suitability techniques for identifying garden locations because it not only identifies potential sites for gardens based on particular criteria, but it also provides a sense of the number of gardens needed to cover a given population of interest (in this case, residents of food deserts).

Location analysis and modeling has been used to support locational decisions in a wide range of applications [66], including emergency service planning [67, 68], school district design [69, 70] and wireless device placement [71] to name a few. Building on the fact that food deserts are demarcated based on distance thresholds, and the goal of the analysis is to service the food desert population, two covering models were considered for this particular study: the location set covering problem [67] and the maximal covering location problem [72]. Different from other types of location models, covering models examine service efficiency using a coverage standard that is often based on travel distance or time: demand is considered covered if it is within the coverage standard of a service provider. Recently, Bao et al. [42] developed a variant of the maximal covering location model to strategically site independent food stores for addressing food desert issues.

In our study, coverage provided by a community garden will be assessed based on whether a food desert is located within the 1-mile travel distance as defined by the USDA. The location set covering model can be used to produce output that would specify the minimum number of gardens needed to ensure that no food desert is left uncovered, while the maximal covering location problem can be used to prescribe the spatial configuration of urban gardens that maximizes the coverage of food deserts when the number of gardens to site is fixed due to a budget constraint.

The model selected to implement in this paper is the maximal location covering problem [72], because it is infeasible to cover all food deserts due to the limited vacant land available. The output of the maximal covering location model is the location of and coverage of food desert residents provided by a given number of gardens. The output from this spatial optimization model also provides geographic information about proposed garden sites, and a tradeoff curve which contains the number of gardens to be sited on the x-axis and the population residing in food deserts covered by the specified number of gardens on the y-axis. From this tradeoff curve, it is possible to understand tradeoffs in the number of gardens located and the percentage of food desert residents covered.

Given the potential for urban gardens to serve as an affordable source of fresh fruits and vegetables for residents in food deserts, the goal of the optimization analysis will be to locate gardens based on two criteria: to cover as many residents in food deserts as possible and to locate these gardens on vacant land within the Phoenix metropolitan area. The location model is specified below.

Maximal covering location problem

1 $$ {\text{Maximize}}\,\sum_{i} w_{i} y_{i} $$

Subject to

2 $$ \sum\limits_{{j \in N_{i} }} {x_{j} } \ge y_{i} \quad \forall i $$

3 $$ \sum\limits_{j} {x_{j} } = p $$

4 $$ x_{j} \in \left\{ {0,1} \right\}\quad \forall j $$

5 $$ y_{i} \in \left\{ {0,1} \right\}\quad \forall i $$

where *i* index of food deserts, *j* index of vacant land, *w*_*i*_ population in food desert *i*

$$ x_{j} = \left\{ {\begin{array}{*{20}l} 1 \hfill &\quad{{\text{if}}\,{\text{vacant}}\,{\text{land}}\,j\,{\text{is}}\,{\text{selected}}\,{\text{for}}\,{\text{the}}\,{\text{conversion}}\,{\text{to}}\,{\text{a}}\,{\text{community}}\,{\text{garden}}} \hfill \\ 0 \hfill &\quad{\text{otherwise}} \hfill \\ \end{array} } \right. $$

$$ y_{i} = \left\{ {\begin{array}{*{20}l} 1 \hfill &\quad {{\text{if}}\,{\text{food desert}}\,i\,{\text{is covered}}} \hfill \\ 0 \hfill &\quad {\text{otherwise}} \hfill \\ \end{array} } \right. $$

*N*_*i*_ = {*j*|*d*_*ij*_ ≤ *D*} consists of all the candidate site *j* that if converted can serve food desert *i* (i.e., the travel distance from *i* to *j* is within *D* the low-access threshold used for defining food deserts). *p*: the number of community gardens to site

Objective (1) aims to maximize the food desert population to be covered. Constraint (2) specifies that a food desert is considered covered only when there is at least one urban garden that is located within the coverage threshold *D*. Given that the food deserts in this study are located in urban areas, we define the coverage threshold *D* to be 1-mile (1.61 km) travel distance in order to be consistent with the definition of food deserts provided by the USDA. Constraint (3) specifies the number of urban gardens to be sited. Constraints (4) and (5) impose binary integer conditions on decision variables *x* and *y* that dictate whether vacant land is selected or not, and whether a food desert is covered or not, respectively.

## Results

**Fig. 2:**
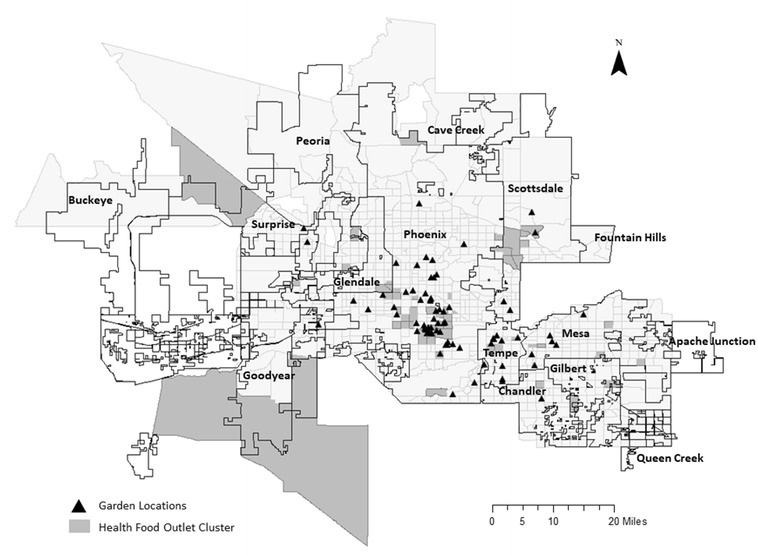
Healthy food outlets and urban garden locations

Before undertaking the spatial optimization analysis to pinpoint proposed garden sites, an analysis of the location of past and present gardens sites is conducted. This portion of the analysis is important because it provides information about the spatial distribution of garden sites, their neighborhood context, and their proximity to food desert locations across the metropolitan area. Figure 2 displays the locations of existing gardens (see Additional file 1 for a shapefile of these gardens). This graphic highlights that the majority of gardens (66%) are located in the city limits of Phoenix in areas that include the historic Encanto district, Maryvale, and South Mountain. Other cities, including Tempe, Mesa, and Chandler, also have garden activity, but the majority is highly centralized in the old urban core. Figure 2 also shows the hotspots of healthy food outlets by census tract, which was produced by aggregating the healthy food outlet point locations from the ESRI Reference USA database to census tracts. The local Moran [73] was used to identify hot-spots of healthy food outlets. These are tracts with a high level of healthy food outlet clustering.^2^

While the figure does not present a formal test of spatial dependence between garden locations and healthy food outlet hotspots, it does provide some support for prior work showing that gardens cluster near healthy food outlets [51]. As the ANOVA results below indicate, these areas are also more likely to be commercial neighborhoods that are zoned to allow retail uses. This means the current locations of gardens do not help residents in food deserts because they are already located in areas with access to healthy food stores. In general, the majority of gardens are located near the central city areas of Phoenix, Tempe, and Mesa. There are also several gardens in the more residential areas of North Phoenix, Scottsdale, and Mesa that are not located near clusters of healthy food outlets, but these are generally the exception.

### Neighborhood context of garden locations

These differences in garden locations raise questions about the neighborhood context of garden sites. To provide some resolution on the extent that neighborhoods with gardens are different from those without gardens, analysis of variance (ANOVA) was conducted to statistically test for neighborhood differences based on five sets of characteristics: demographics, socio-economic status, land use characteristics, housing type, food outlet type, and commuting characteristics. Given the relatively low number of garden-containing census tracts (75 out of 880 in the study area, or 8.5% of tracts), garden-adjacent census tracts were also included in the analysis (34.3% of tracts) in order to evaluate the neighborhood context of communities with gardens. Garden-adjacent tracts are also important to identify since they are closer to garden locations—and thus more likely to receive some supplementary benefit—than other tracts in the metropolitan area.

Table 3 presents summary results of this analysis and highlights significant differences between census tracts with gardens, tracts adjacent to those with gardens, and tracts without gardens. Detailed ANOVA results may be found in Additional file 2 included at the end of this paper. In terms of interpreting the information in Table 3, each variable is listed next to the tract type with the *highest value* of that variable; for example, industrial, neighborhood commercial, educational, office, and medical land uses are all statistically different between the tract types, *and* have higher percentages in garden-containing tracts. Similarly, garden-adjacent tracts show the highest percentage of multi-family residential land use. In terms of demographics, urban garden tracts and tracts adjacent to gardens are more racially and ethnically diverse; tracts with gardens have a higher percentage of Black and Hispanic residents than do non-garden tracts. They also have lower levels of educational attainment. In terms of other measures of socio-economic status, garden tracts and tracts adjacent to gardens have a higher percentage of persons who are unemployed, on food stamps, and without healthcare.

**Table 3:** Highest values of various characteristics for no garden, garden-adjacent, and garden-containing tracts

Tract type	Land use characteristics	Food deserts	Housing	Socio-demographics	Food outlets	Urban design and transportation
Contains garden	Industrial	Low access low income share at 1/2-mile (0.8 km)	% Vacant housing units	% Black	# Supermarkets	
	Neighborhood commercial			% Hispanic	# Convenience outlets	
	Educational			% Food stamps	# Bakeries	
	Office				# Restaurants	
	Medical				# Other grocery outlets	% Drove alone to work
					# Fruit and vegetable outlets	% Non-auto commuters
						% < 15 min commute
						% ≥ 30 min commute
Garden-adjacent	Multi-family residential				# Specialty food outlets	
					# Meat and fish outlets	
No gardens	Single-family residential low density	Low access kids’ share at 1/2-mile (0.8 km)	Median home value	% Bachelor’s		
	Single-family residential medium density		% Owner occupied			
	Single-family residential high density					
	Developable agriculture					
	Developable land					
	Developing residential					

Table shows only results significant at the 10% level or better

Aside from demographic and socio-economic differences, there are also interesting differences in land uses amongst the three categories of tracts analyzed, particularly for tracts with gardens and tracts adjacent to garden tracts. These tracts have less land dedicated to residential land, but more land area dedicated to medical, office, and educational uses than tracts without gardens. As for the characteristics of nearby food outlets, gardens and tracts neighboring garden tracts have higher access to a variety of food outlets including restaurants, supermarkets, and convenience stores. Interestingly tracts with gardens also had the lowest share of workers commuting to work by driving alone, the highest share of workers commuting by non-auto modes (transit, walking and cycling), the highest percentage of residents with a commute under 15 min, and the lowest percentage of residents with a commute of 30 min of more.

**Fig. 3:**
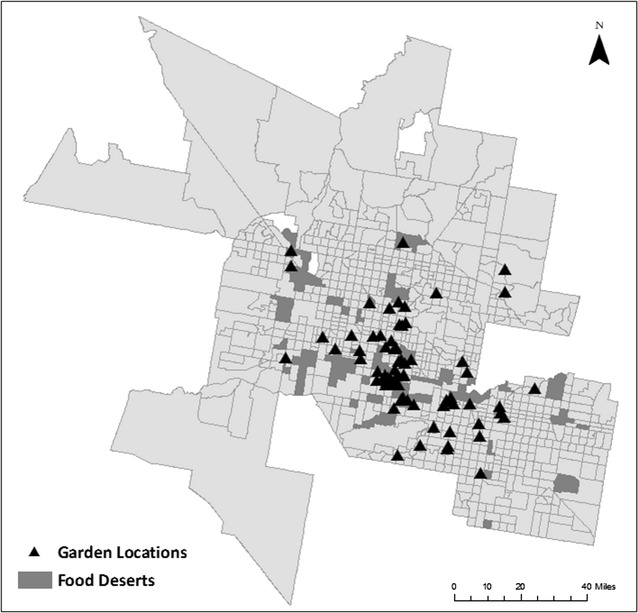
Urban garden and food desert locations

Figure 3 displays the locations of gardens and food deserts in the metropolitan area. It highlights that many gardens are not located in food deserts; in fact, only 24 out of the 75 gardens (32%) are located in food desert tracts. Also, of the 68 active urban gardens identified at the time of this analysis, only nine cover food deserts with a population of 27,290, corresponding to just 8.4% of all food desert residents. Several of the uncovered food deserts are located in exurban locations to the West of downtown Phoenix in neighborhoods such as El Mirage and Glendale. Uncovered food deserts are also evident in the east of the metropolitan area in Mesa. Based on this distribution of gardens, it appears future garden sites could be located more strategically to cover residents in food desert locations.

### Siting urban gardens

To analyze how gardens could be distributed better, a maximal covering spatial optimization model was used to identify gardens sites to provide better coverage for food desert residents. To do this, only vacant land classified as developable was considered; military and native community lands were excluded. Land considered too small for community gardens (< 5000 ft^2^) was also excluded. This threshold of 5000 ft^2^ is based on recommendations that to achieve a critical mass of gardeners, the total size of a garden should be a minimum of 3000–3500 ft^2^ so that it may contain 10–12 good sized garden plots [74]. A size of 5000 ft^2^ would accommodate this number of plots and also provides space for a toolshed and community garden activities.

The analysis resulted in 5947 pieces of vacant land selected to serve as potential urban garden sites. The coverage assessment was performed based on the travel distance from a food desert to a candidate garden site using ESRI’s Network Analyst and the region’s street network. During the distance calculation, vacant land was represented using the geometric centroids and food deserts were converted to points using their population centers. The maximal covering location problem introduced in the previous section was then solved to identify which vacant land sites can serve the food deserts not served currently by existing gardens.

**Fig. 4:**
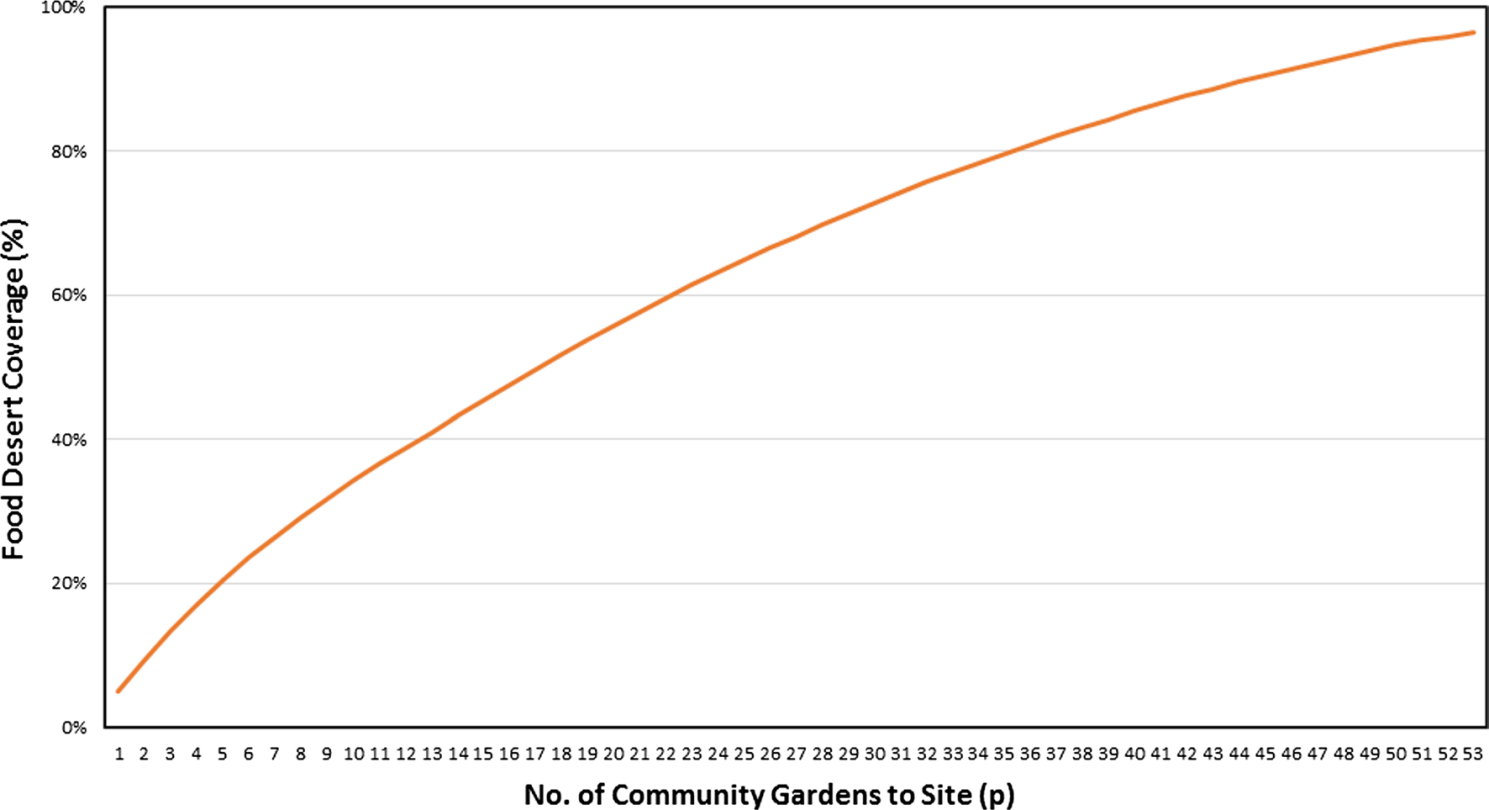
Tradeoff curve of garden numbers and food desert coverage

**Fig. 5:**
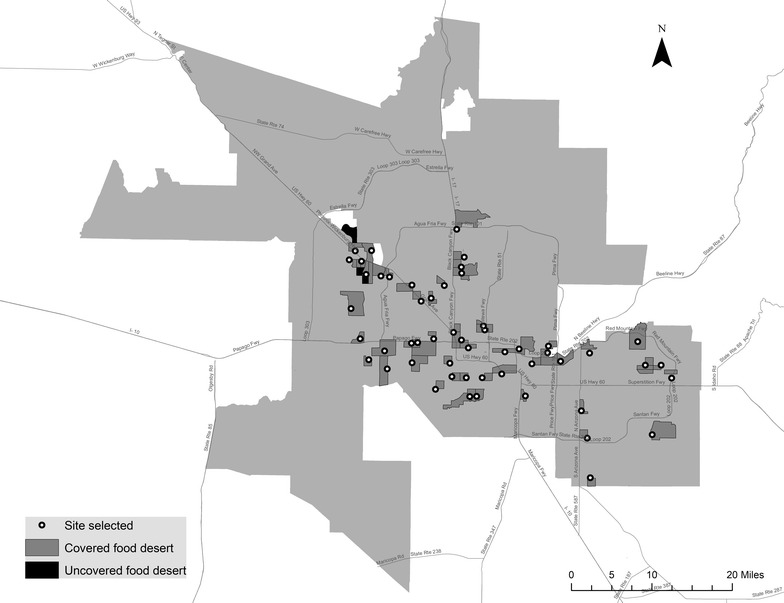
Proposed garden sites and food desert locations

Figure 4 presents a tradeoff curve that summarizes the results of this analysis. On the x-axis of this graph is the number of gardens, and the y-axis represents the percentage of food desert residents covered by siting *p* number of community gardens. The tradeoff curve provides important insights for planners and government agencies to better allocate limited funds for food project planning. Similar to many other maximal coverage location problem applications, marginal coverage achieved decreases with the number of facilities sited. For example, siting 25 urban gardens achieves coverage of about 65% of the food desert population whereas an increase of gardens to twice that number (50 gardens), achieves 30% more coverage. Constrained by the location of the vacant land available, it is infeasible to achieve complete coverage of all 68 food deserts not covered by existing gardens. This is because three food deserts are left uncovered due to the lack of available land closer to food desert sites. The best coverage possible can be obtained by siting 53 urban gardens, providing maximal coverage of 65 food deserts with 96.4% of the food desert population covered (Additional file 3). This is a vast improvement over the current distribution of gardens; the 68 active community garden sites only cover 8.4% of food desert populations. A map of the 53 proposed garden sites along with food desert locations is shown in Fig. 5. Several of the proposed sites (45%) are located in the city limits of Phoenix. Proposed garden sites to the west of Phoenix include the communities of El Mirage, Glendale, Sun City, and Peoria. To the southeast of Phoenix, other proposed garden sites are located in Tempe, Chandler, and Mesa.

## Discussion

Across the world, urbanization continues at a rapid pace. As agricultural land is converted to other uses and people become disconnected from traditional food sources, access to healthy food is a growing issue for urban residents worldwide. Given the health implications associated with the lack of access to healthy food [9, 75, 76], this study set out to demonstrate how spatial optimization models may be used to better locate urban gardens to improve access for residents and to resolve the issue of unused vacant land simultaneously. This technique is demonstrated here for the Phoenix Arizona metropolitan area but can also be applied to any city globally where food access and vacant land issues are present. As mentioned previously, several cities in countries around the globe, such as Afghanistan, India, and Brazil, are currently experiencing similar problems associated with rapid rates of urbanization.

Analytical results reveal important demographic, socio-economic, and land use differences between tracts with or near urban gardens and tract without or not near urban gardens. Tracts with or near gardens are more racially and ethnically diverse and also contain characteristics of low socio-economic status such as lower levels of educational attainment and higher rates of unemployment compared to non-garden tracts. These results are encouraging because they indicate that residents perhaps *most* in need of healthy food are often within close proximity to urban gardening activity. Unfortunately, an analysis of the spatial distribution of food deserts and urban gardens reveals that the distribution of urban gardens at the time of this analysis covered less than 10% of food desert residents, which highlights that an alternative distribution of urban gardening activity would improve access to these sources of fresh fruits and vegetables. Spatial optimization models are used to suggest alternative locations of urban gardens using vacant land. These model results suggest an alternative arrangement of 53 gardens that would provide coverage of 96.4% of the food desert population.

That said, it is important to note some limitations of this analysis. First, there are additional considerations beyond the availability of land and lack of food access that will need to be investigated further in the proposed garden sites. One of these considerations is the quality of soil, which prior work has noted is a potential issue for urban gardening activity [77, 78]. Thus, it is recommended that the soil quality in the proposed sites be tested for contaminants before planting commences. A second consideration is the potential volume of food that could be produced at garden sites. Prior studies have noted that the food production capacity of urban gardens may be insufficient to provide food in the necessary quantities needed [51]. However, other studies have noted that coordinated planning efforts to foster urban gardening activity can produce a large proportion of local food needs [79]. To account for this concern, the gardens sited in this analysis ensure that at least 5000 ft^2^ are used for gardening activity. However, additional steps will need to be taken from a garden management perspective to ensure proper crop rotation and to ensure that the volume of fruits and vegetables grown is as such, that it may serve as a good supply of healthy foods for garden participants and the surrounding community. Third, once established, a concentrated and enduring effort to maintain urban gardens sites is needed to preserve these spaces. Gardens are a notoriously transient urban activity [49] and preservation plans are needed so as not to upend activity once it is commenced. This was the case with a large urban garden started as part of the Phoenix Renews project, which was shut down due to financial issues with the land on which the garden was placed [64]. Fourth, although citing gardens can reduce the physical distance to food, it may not reduce the temporal distance. Low-income people are more likely to be multiple job holders and may lack the time and also the knowledge to cook fresh vegetables. Finally, it is important to note that the mere provision of *access* to fresh fruits and vegetables is not enough to resolve dietary problems and the health issues stemming from poor diets. Studies of the built environment and health have uncovered a range of factors that influence obesity from land-use mix, crime, type of food outlets present, and urban design that is pedestrian oriented [80]. Thus, increasing access to urban gardens is just the first step to improving healthy food consumption for people. Access needs to be coupled with education efforts about the health value of fruits and vegetables grown in the gardens, as well as promotion of the gardens themselves to encourage participation by area residents. The pricing of any products sold should also be as such, that they are affordable to folks in a wide-variety of income strata. Recipes can also be provided that would educate purchasers of products about the preparation of fruits and vegetables to improve health outcomes.

## Conclusion

As rapid urbanization continues globally so too are issues of food access and vacant land likely to become more prevalent. To combat these related issues, more sophisticated planning strategies are needed to improve food access for residents. Although enhancing access is just the first step in improving healthy food consumption, urban gardens represent an inexpensive way to provide food to nearby residents. As demonstrated in this paper, spatial optimization models are an analytical tool that can be used to strategically locate these food sources on unused urban land, thereby mitigating two problems evident in rapidly expanding cities around the world.

## Additional files

**Additional file 1.** Past and Present Phoenix Garden Locations. Point shapefile of the garden data used in this analysis.

**Additional file 2.** Results of ANOVA tests for no garden, garden-adjacent, and garden-containing tracts. Three tables showing results of ANOVA analysis for each of the garden types and each of the variables of interest. Table includes the mean, standard deviation, and statistical significance for each variable/tract-type combination.

**Additional file 3.** Proposed Phoenix garden locations. Point shapefile of garden data generated from the spatial optimization analysis.

## Abbreviations

ACGA: American Community Garden Association
ACS: American Community Survey
ANOVA: analysis of variance
GIS: geographic information systems
ESRI: Environmental Systems Research Institute
MAG: Maricopa Association of Governments
MCCE: Maricopa County Cooperative Extension
NHGIS: National Historic Geographic Information System
RUCA: Rural–Urban Commuting Area
USDA: United States Department of Agriculture
WHO: World Health Organization

## Footnotes

1 Note this definition of healthy food outlets is more comprehensive than that of the USDA, which bases its definition of food deserts on access to supermarkets [39].

2 Hotspots are defined as tracts corresponding to the high–high and high-low output of the local Moran. Census tracts are drawn to include roughly 4000 people [81]; thus, mapping a density measure or per capita number of food outlets in Fig. 1 would be redundant.
