# Supplementary material for: Gardening in the desert: a spatial optimization approach to locating gardens in rapidly expanding urban environments
Source: Int J Health Geogr. 2017 Oct 16;16:37. doi: 10.1186/s12942-017-0110-z (PMC5644113; doi:10.1186/s12942-017-0110-z)
Supplement: Supplementary file 2 — Additional file 2. Results of ANOVA tests for no garden, garden-adjacent, and garden-containing tracts. Three tables showing results of ANOVA analysis for each of the garden types and each of the variables of interest. Table includes the mean, standard deviation, and statistical significance for each variable/tract-type combination. [file 12942_2017_110_MOESM2_ESM.docx]

**Results of ANOVA Tests for No Garden, Garden-Adjacent, and Garden-Containing Tracts.**

| *Land Use Characteristics* |  | *No Garden* | *Garden-Adjacent* | *Contains Garden* | *Sig.* |
| --- | --- | --- | --- | --- | --- |
| **Industrial** | Mean | 0.035 | 0.071 | 0.091 | *** |
|  | Std. Dev. | 0.177 | 0.233 | 0.223 |  |
| **Single-Family Residential Low Density** | Mean | 0.176 | 0.039 | 0.015 | *** |
|  | Std. Dev. | 0.712 | 0.216 | 0.051 |  |
| **Single-Family Residential Medium Density** | Mean | 0.122 | 0.082 | 0.075 | ** |
|  | Std. Dev. | 0.217 | 0.205 | 0.154 |  |
| **Single-Family Residential High Density** | Mean | 0.333 | 0.237 | 0.217 | *** |
|  | Std. Dev. | 0.276 | 0.228 | 0.213 |  |
| **Neighborhood Commercial** | Mean | 0.039 | 0.043 | 0.059 | ** |
|  | Std. Dev. | 0.054 | 0.064 | 0.070 |  |
| **Regional Commercial** | Mean | 0.017 | 0.016 | 0.009 |  |
|  | Std. Dev. | 0.043 | 0.045 | 0.027 |  |
| **Multi-Family Residential** | Mean | 0.038 | 0.064 | 0.060 | *** |
|  | Std. Dev. | 0.052 | 0.074 | 0.051 |  |
| **Developable Agriculture** | Mean | 0.147 | 0.024 | 0.013 | *** |
|  | Std. Dev. | 0.632 | 0.127 | 0.091 |  |
| **Developable Land** | Mean | 0.851 | 0.119 | 0.089 | *** |
|  | Std. Dev. | 4.776 | 0.398 | 0.187 |  |
| **Educational** | Mean | 0.034 | 0.026 | 0.045 | ** |
|  | Std. Dev. | 0.060 | 0.054 | 0.067 |  |
| **Office** | Mean | 0.009 | 0.021 | 0.027 | *** |
|  | Std. Dev. | 0.018 | 0.050 | 0.055 |  |
| **Medical** | Mean | 0.007 | 0.008 | 0.013 | * |
|  | Std. Dev. | 0.021 | 0.023 | 0.024 |  |
| **Developing Residential** | Mean | 0.031 | 0.005 | 0.005 | *** |
|  | Std. Dev. | 0.140 | 0.022 | 0.029 |  |
| **Public Special Events** | Mean | 0.031 | 0.023 | 0.035 |  |
|  | Std. Dev. | 0.136 | 0.058 | 0.104 |  |
| **Religious Institutions** | Mean | 0.011 | 0.012 | 0.016 |  |
|  | Std. Dev. | 0.017 | 0.019 | 0.013 |  |
| **Vacant Developable** | Mean | 0.671 | 0.089 | 0.070 | ** |
|  | Std. Dev. | 4.576 | 0.346 | 0.119 |  |

| *Food Deserts* |  | *No Garden* | *Garden-Adjacent* | *Contains Garden* | *Sig.* |
| --- | --- | --- | --- | --- | --- |
| **Low access low income share at 1/2-mile** | Mean | 0.158 | 0.206 | 0.307 | *** |
|  | Std. Dev. | 0.134 | 0.174 | 0.245 |  |
| **Low access kids share at 1/2-mile** | Mean | 0.685 | 0.578 | 0.605 | *** |
|  | Std. Dev. | 0.283 | 0.326 | 0.318 |  |
| **Low access senior share at 1/2-mile** | Mean | 358.406 | 289.451 | 225.480 |  |
|  | Std. Dev. | 467.864 | 423.513 | 203.554 |  |
| *Housing Characteristics* |  |  |  |  |  |
| **Median Home Value** | Mean | 207249.120 | 167425.490 | 157610.670 | *** |
|  | Std. Dev. | 144577.580 | 118164.430 | 122711.750 |  |
| **Percent Owner Occupied** | Mean | 0.672 | 0.537 | 0.448 | *** |
|  | Std. Dev. | 0.206 | 0.214 | 0.237 |  |
| **Percent Vacant Housing Units** | Mean | 0.124 | 0.137 | 0.149 | *** |
|  | Std. Dev. | 0.079 | 0.079 | 0.071 |  |
| **Median Contract Rent** | Mean | 988.608 | 842.149 | 736.760 |  |
|  | Std. Dev. | 355.663 | 294.535 | 293.953 |  |
| *Demographics and Socio-Economic Status* |  |  |  |  |  |
| **Percent Black** | Mean | 0.044 | 0.053 | 0.073 | *** |
|  | Std. Dev. | 0.050 | 0.052 | 0.074 |  |
| **Percent Hispanic** | Mean | 0.237 | 0.355 | 0.435 | *** |
|  | Std. Dev. | 0.201 | 0.265 | 0.269 |  |
| **Percent Bachelor's** | Mean | 0.203 | 0.174 | 0.160 | *** |
|  | Std. Dev. | 0.099 | 0.104 | 0.117 |  |
| **Percent Food Stamps** | Mean | 0.101 | 0.156 | 0.226 | *** |
|  | Std. Dev. | 0.101 | 0.124 | 0.170 |  |
| **Percent No Healthcare** | Mean | 0.132 | 0.199 | 0.236 |  |
|  | Std. Dev. | 0.092 | 0.114 | 0.117 |  |
| **Percent Under 18 With No Healthcare** | Mean | 0.023 | 0.033 | 0.039 |  |
|  | Std. Dev. | 0.022 | 0.030 | 0.030 |  |
| **Percent Unemployment** | Mean | 0.085 | 0.096 | 0.122 |  |
|  | Std. Dev. | 0.063 | 0.048 | 0.065 |  |

| *Food Outlets* |  | *No Garden* | *Garden-Adjacent* | *Contains Garden* | *Sig.* |
| --- | --- | --- | --- | --- | --- |
| **Number Candy and Nut Outlets** | Mean | 0.064 | 0.096 | 0.093 |  |
|  | Std. Dev. | 0.322 | 0.408 | 0.293 |  |
| **Number Supermarkets** | Mean | 0.583 | 0.887 | 1.267 | *** |
|  | Std. Dev. | 0.779 | 1.041 | 1.359 |  |
| **Number Convenience Outlets** | Mean | 0.563 | 1.020 | 1.267 | *** |
|  | Std. Dev. | 0.854 | 1.093 | 1.189 |  |
| **Number Bakeries** | Mean | 0.282 | 0.427 | 0.533 | *** |
|  | Std. Dev. | 0.647 | 0.786 | 0.905 |  |
| **Number Specialty Food Outlets** | Mean | 0.022 | 0.053 | 0.040 | * |
|  | Std. Dev. | 0.159 | 0.239 | 0.197 |  |
| **Number Restaurants** | Mean | 6.127 | 8.616 | 11.173 | *** |
|  | Std. Dev. | 7.190 | 9.827 | 14.196 |  |
| **Number Natural Food Outlets** | Mean | 0.179 | 0.232 | 0.187 |  |
|  | Std. Dev. | 0.455 | 0.558 | 0.456 |  |
| **Number Meat and Fish Outlets** | Mean | 0.054 | 0.152 | 0.107 | *** |
|  | Std. Dev. | 0.251 | 0.420 | 0.352 |  |
| **Number Other Grocery Outlets** | Mean | 0.000 | 0.000 | 0.013 | *** |
|  | Std. Dev. | 0.000 | 0.000 | 0.115 |  |
| **Number Fruit and Vegetable Outlets** | Mean | 0.070 | 0.109 | 0.360 | *** |
|  | Std. Dev. | 0.284 | 0.371 | 1.061 |  |
| **Number Dairy Outlets** | Mean | 0.042 | 0.046 | 0.053 |  |
|  | Std. Dev. | 0.210 | 0.266 | 0.280 |  |
